# Effects of SARS-CoV-2 on Cardiovascular System: The Dual Role of Angiotensin-Converting Enzyme 2 (ACE2) as the Virus Receptor and Homeostasis Regulator-Review

**DOI:** 10.3390/ijms22094526

**Published:** 2021-04-26

**Authors:** Aneta Aleksova, Giulia Gagno, Gianfranco Sinagra, Antonio Paolo Beltrami, Milijana Janjusevic, Giuseppe Ippolito, Alimuddin Zumla, Alessandra Lucia Fluca, Federico Ferro

**Affiliations:** 1 Cardiothoracovascular Department, Azienda Sanitaria Universitaria Giuliano Isontina (ASUGI) and Department of Medical Surgical and Health Science, University of Trieste, 34149 Trieste, Italy; gagnogiulia@gmail.com (G.G.); gianfranco.sinagra@asugi.sanita.fvg.it (G.S.); mjanjusevic@units.it (M.J.); alessandralucia.fluca@units.it (A.L.F.); fferro@units.it (F.F.); 2 Department of Medicine (DAME), University of Udine, 33100 Udine, Italy; antonio.beltrami@uniud.it; 3 National Institute for Infectious Diseases Lazzaro Spallanzani-IRCCS, 00135 Rome, Italy; giuseppe.ippolito@inmi.it; 4 Department of Infection, Division of Infection and Immunity, Centre for Clinical Microbiology, University College London, London NW3 2PF, UK; a.zumla@ucl.ac.uk; 5 National Institute for Health Research Biomedical Research Centre, University College London Hospitals, London NW1 2BU, UK

**Keywords:** cardiovascular system, ACE2, RAS, COVID-19, SARS- CoV-2, TMPRSS2, ADAM17, pandemic, vaccines

## Abstract

Angiotensin-converting enzyme 2 (ACE2) is the entry receptor for severe acute respiratory syndrome coronavirus-2 (SARS-CoV-2), the cause of Coronavirus Disease-2019 (COVID-19) in humans. ACE-2 is a type I transmembrane metallocarboxypeptidase expressed in vascular endothelial cells, alveolar type 2 lung epithelial cells, renal tubular epithelium, Leydig cells in testes and gastrointestinal tract. ACE2 mediates the interaction between host cells and SARS-CoV-2 spike (S) protein. However, ACE2 is not only a SARS-CoV-2 receptor, but it has also an important homeostatic function regulating renin-angiotensin system (RAS), which is pivotal for both the cardiovascular and immune systems. Therefore, ACE2 is the key link between SARS-CoV-2 infection, cardiovascular diseases (CVDs) and immune response. Susceptibility to SARS-CoV-2 seems to be tightly associated with ACE2 availability, which in turn is determined by genetics, age, gender and comorbidities. Severe COVID-19 is due to an uncontrolled and excessive immune response, which leads to acute respiratory distress syndrome (ARDS) and multi-organ failure. In spite of a lower ACE2 expression on cells surface, patients with CVDs have a higher COVID-19 mortality rate, which is likely driven by the imbalance between ADAM metallopeptidase domain 17 (ADAM17) protein (which is required for cleavage of ACE-2 ectodomain resulting in increased ACE2 shedding), and TMPRSS2 (which is required for spike glycoprotein priming). To date, ACE inhibitors and Angiotensin II Receptor Blockers (ARBs) treatment interruption in patients with chronic comorbidities appears unjustified. The rollout of COVID-19 vaccines provides opportunities to study the effects of different COVID-19 vaccines on ACE2 in patients on treatment with ACEi/ARB.

## 1. Introduction

Severe acute respiratory syndrome coronavirus 2 (SARS-CoV-2) was first identified as a novel human pathogen in December 2019 and since has caused a worldwide pandemic [1]. As of 8 February 2021, there have been over 105 million Coronavirus Disease-2019 (COVID-19) cases including 2.3 million deaths reported by the World Health Organization (WHO) [2]. Epidemiologic studies highlight that age, gender and comorbidities (hypertension, renal insufficiency, diabetes and ischemic heart disease) are frequently associated with greater mortality risk after SARS-CoV-2 infection [3,4].

The pathogenesis of COVID-19 has two stages [5,6,7]: the first one, where SARS-CoV-2 replicates and patients manifest a range of non-specific symptoms (e.g., fever, muscle aches, shortness of breath, headache, sore throat and gastrointestinal discomfort) [3,6]. The second stage is characterized by an adaptive immune response with humoral, cellular and cytokine responses manifesting in a large range of clinical presentations [6]. Some patients develop the most severe forms of COVID-19, leading to fatal complications such are acute respiratory distress syndrome (ARDS), acute kidney injury and thromboembolism [3,6].

Angiotensin-converting enzyme 2 (ACE2) is the entry receptor for SARS-CoV-2, which is the cause of COVID-19 in humans. ACE2 is a type I transmembrane metallocarboxypeptidase expressed in endothelial cells, alveolar type 2 lung epithelial cells, renal tubular epithelium, Leydig cells in the testes, and gastrointestinal tract [7]. ACE2 mediates the interaction between host cells and SARS-CoV-2 spike (S) protein [8]. However, ACE2 is not only a SARS-CoV-2 receptor. Indeed, it also has an important homeostatic function regulating renin-angiotensin system (RAS), which is pivotal for both the cardiovascular and immune systems [9]. Therefore, ACE2 appears to be the key link between SARS-CoV-2 infection, cardiovascular diseases (CVDs) and immune response [8,10,11]. RAS pathway has a fundamental role in human body homeostasis and its imbalance is associated with inflammation and cardiovascular alterations [12,13]. SARS-CoV-2 binds to human cells through ACE2, and it appears that pre-existing alterations in ACE2 expression and activity could determine susceptibility to SARS-CoV-2 infections [4,14].

The aim of this review is to summarize the state of the art in ACE2-SARS-CoV-2 interactions in the context of the cardiovascular system and to discuss the implications and impact of the use of ACE inhibitors (ACEi) and angiotensin receptor blockers (ARB) in patients with SARS-CoV-2 infection. We also discuss the rollout of COVID-19 vaccines and the opportunities this provides to study the effects of different COVID-19 vaccines on ACE2 in patients on treatment with ACEi/ARB.

## 2. ACE2 Physiological Role

The ACE2 gene, located on chromosome Xp22, consists of 18 exons and 20 introns [15] and encodes a type I transmembrane glycoprotein of 805 amino acids [16,17]. The N-terminal catalytically active domain is located in the extracellular space while the intracellular C-terminal consists of a collectin and an insulin-like domain [16,17].

In RAS, Renin produces Angiotensin I (Ang I) starting from Angiotensinogen, then angiotensin-converting enzyme (ACE) removes two amino acids from Ang I generating the active peptide Angiotensin II (Ang II) [18]. In the classical RAS, Ang II binds to angiotensin type 1 and 2 receptors (AT1R e AT2R) promoting vasoconstriction, inflammation, increase of blood pressure and myocardial contraction [18]. ACE2 acts in a different way: specifically, it generates Angiotensin 1-7 (Ang 1-7) after Ang II cleavage [18,19]. Subsequently, Ang 1-7 is responsible for vasodilatation and for the production of anti-inflammatory molecules by interacting with Mas receptor (MasR) [18]. Furthermore, ACE2 acts on Ang I, forming Angiotensin 1-9 (Ang 1-9), thus contributing to the reduction of Ang I availability for classical RAS [13].

ACE2 undergoes proteolytic cleavage at different sites by both the type II transmembrane serine protease (TMPRSS2) and ADAM Metallopeptidase Domain 17 (ADAM17) [20,21,22]. Specifically, ADAM17 activity on ACE2 generates an extracellular soluble ACE2 (sACE2) fragment (i.e., shedding) [20]. A recent study demonstrated that, after shedding, the remaining transmembrane fragment is targeted by γ-Secretase generating an intracellular domain (ICD) [23]. Preliminary analyses suggest that ICD is not involved in the regulation of ACE2, TMPRSS2 or ADAM17 expression [23]. The ADAM17-mediated shedding is a constitutive mechanism that is suppressed by TMPRSS2 activity [24,25].

## 3. ACE2 Balance and SARS-COV-2 Infection

**Figure 1:**
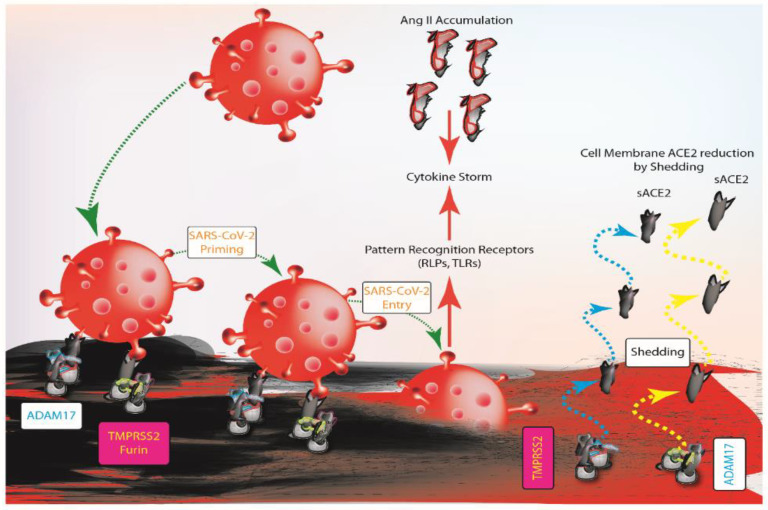
Schematic representation of host cell-SARS-CoV-2 interaction during infection before the interaction between virus and host cell, furin and TMPRSS2 cut the spike (S) protein. The process allows the interaction between S protein and ACE2, which triggers the innate immune response. Ang II contributes to the inflammation sustaining the production of cytokines. ADAM17 and TMPRSS2 mediate the shedding process producing the soluble form of ACE2 thus influencing ACE2 availability on the cell surface and the interaction with SARS-CoV-2. ADAM17, ADAM metallopeptidase domain 17; TMPRSS2, type II transmembrane serine protease; sACE2, soluble ACE2; Ang II, angiotensin II; RLRs, RIG-like receptors; TLRs, toll-like receptors.

SARS-CoV-2 interacts with host cells through the surface spike (S) protein [26]. Initially, S protein undergoes activation after proteolytic cleavage by furin and TMPRSS2 (i.e., priming) on the cell surface [26]. This cleavage allows the separation between S1 and S2 subunits of S protein [27]. As a result, the S2 subunit rearranges permitting the interaction with ACE2 and the subsequent endocytosis of viral particles [26,28]. Evidence of ACE2 cleavage by both TMPRSS2 and ADAM17, in physiological conditions and in SARS-CoV-2 infection, provides new insights for greater complexity in virus-host interaction [24]. Specifically, ACE2-S protein interaction increases ADAM17 activity [16]. The increase of ACE2 shedding reduces its availability on the cell surface and it also leads to the accumulation of Ang II, which in turn generates positive feedback for ACE2 shedding by enhancing ADAM17 activity [14,20]. At first sight, the reduced availability of ACE2, due to ADAM17 shedding, could be evaluated as a protective mechanism against SARS-CoV-2. However, ADAM17 activity could not be considered fully beneficial to counteract SARS-CoV-2 infection. In fact, it is still questionable whether SARS-CoV-2 trapping by sACE2 could promote the clearance of viral particles and prevent them from being internalized [27,28]. Nonetheless, it is likely that the protective effect of sACE2 is neutralized in case of high viral load. Furthermore, evidence suggests that ACE2 shedding could facilitate viral entry [16]. After viral infection, the innate immune system drives initial response via pattern recognition receptors (PRRs), as RIG-like receptors (RLRs) and Toll-like receptors (TLRs), mediating inflammation [29]. It has been documented that TLRs could activate ADAM17 leading to tumor necrosis factor alpha (TNF-α), interleukin-6 (IL-6) and epidermal growth factor receptor (EGFR) after viral infection [14,30]. Furthermore, because the role of ACE2 is not limited to being a viral receptor [18], the Ang II accumulation causes the massive release of cytokines via AT1R [16]. Therefore, the “cytokine storm” triggers an uncontrolled immune response and tissue damage [16,29] (Figure 1).

## 4. Does ACE2 Influence Probability of SARS-COV-2 Infection and Worse Outcome?

There is great interest in factors that affect immune responses and increase susceptibility toward SARS-CoV-2 infection because they could be of great help in predicting patient outcomes. As described for other viral infections, part of the inter-individual susceptibility is determined by genetic variants [31]. “The COVID Human Genetic Effort” consortium has been formed to identify monogenic variants for resistance or susceptibility to SARS-CoV-2 [31] and study the differences of allele frequency among ethnic groups to explain the different susceptibility among various populations [31,32]. Analysis of ACE2, TMPRSS2 and ADAM17 genes is of particular interest because variants may influence the probability of cell–virus interaction and host response against SARS-CoV-2 infection [33]. In particular, the ACE2 gene exhibits high variability and up to date, more than 1700 variants have been described [15]. Some single nucleotide polymorphisms (SNPs) could influence ACE2 gene expression, activity and interaction with the S protein thus affecting its binding affinity to SARS-CoV-2 [15,33,34,35,36]. Furthermore, several TMPRSS2 and ADAM17 polymorphisms have been identified and it has been demonstrated that some of these variants have higher expression and activity, thus influencing susceptibility toward SARS-CoV-2 infection [15,34,35]. Therefore, the association of polymorphisms with COVID-19 severity needs further investigation [15,35]. 

Along with genetic factors, habits such as cigarette smoking and diet seem to have a significant effect on ACE2. Nicotine appears to promote classical RAS accounting for cardiovascular complications and reduction of immune response efficiency [37,38], and the expression of nicotinic acetylcholine receptors in airway cells may promote SARS-CoV-2 uptake through nicotine-mediated cellular signaling [39]. Consequently, smoking may influence susceptibility and therefore explain why many individuals with severe COVID-19 are also smokers [37,40,41,42]. In addition, salt- and glucose-rich diets may also influence ACE2 expression and activity, increasing the risk of RAS imbalance and SARS-CoV-2 susceptibility [14].

**Figure 2:**
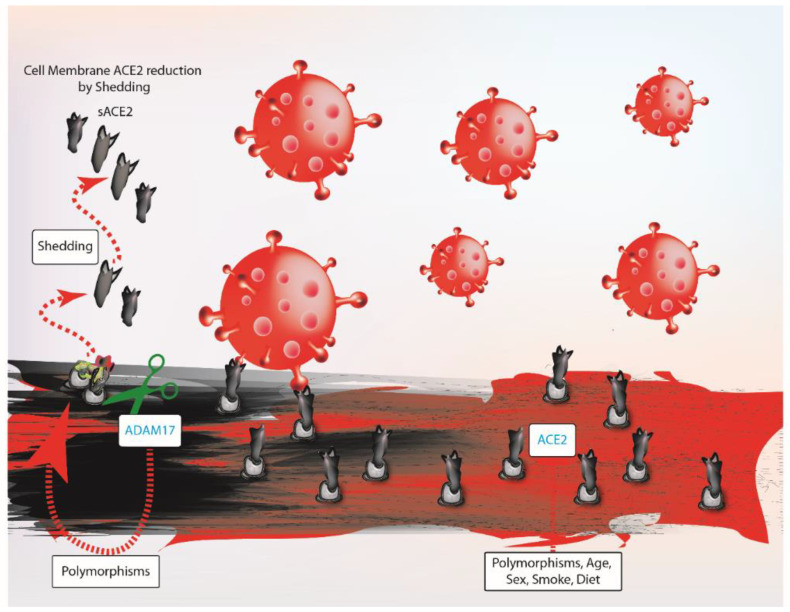
Schematic representation of ADAM17 activity, which efficiency depends on its polymorphisms. Differences between individuals for ACE2 are influenced by genetics factors coupled with habits such as smoking and diet. Therefore, the reduction of ACE2 availability on cell surface could explain variations in susceptibility among populations. ADAM17, ADAM metallopeptidase domain 17; ACE2, angiotensin-converting enzyme 2; sACE2, soluble ACE2.

Studies are required to determine how age, gender and comorbidities influence ACE2 levels on the cell surface [4,14]. ACE2 expression is known to decrease with age and its reduction is stronger in males than females [43,44]. Because the ACE2 locus on the X-chromosome is only partially inactivated, in theory, females have double the dose of the ACE2 gene with respect to males [45]. The discrepancy in ACE2 expression between genders could be also explained by hormonal factors [46,47]. Specifically, the decline of sex hormones with age could contribute to reduced ACE2 expression thus influencing SARS-CoV-2 susceptibility [43,46,47]. Furthermore, ACE2 shedding shows differences according to age and gender [48]. In line with this, preliminary observations propose that TMPRSS2 and ADAM17 transcription is also affected by sex hormones [15,22]. These data suggest that the association between age, gender and shedding needs further investigations. Furthermore, gender differences in susceptibility towards SARS-CoV-2 might be related to the immune response. In fact, many immune-associated genes (e.g., TLRs, RLRs) have an X-linked expression pattern, which is activated by hormones [45]. Specifically, estrogens have immuno-stimulant effects, while androgens are immunosuppressive [45]. Taken together, it is likely that the increase in ACE2 expression, reduced ACE2 cleavage and the more efficient immune response could contribute to lower SARS-CoV-2 susceptibility in females (Figure 2).

**Figure 3:**
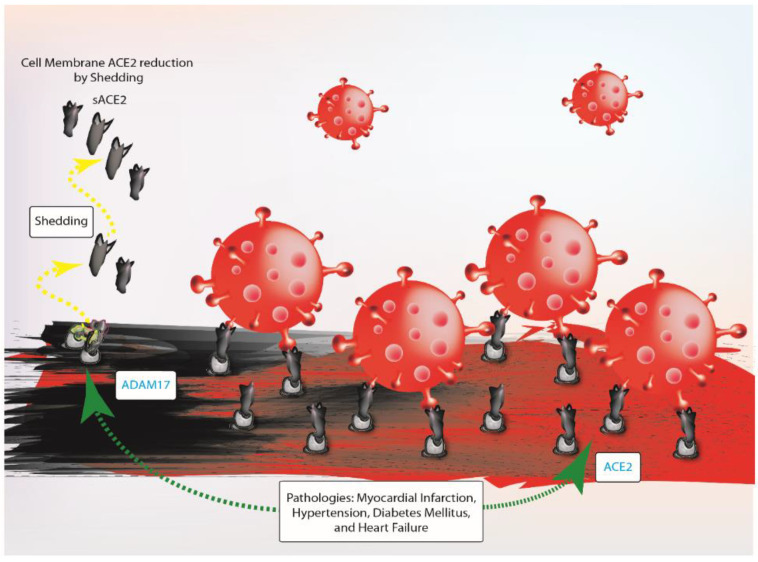
Pathologies such as myocardial infarction, hypertension, diabetes mellitus and heart failure increase the availability of ACE2 suggesting that comorbidities cause high susceptibility towards SARS-CoV-2. ADAM17, ADAM metallopeptidase domain 17; ACE2, angiotensin-converting enzyme 2; sACE2, soluble ACE2.

Notably, although ACE2 has positive effects, its expression in human tissues exacerbates pathologies such as myocardial infarction, hypertension, diabetes mellitus and heart failure [49,50]. The reason for this discrepancy could be explained by the enhanced ADAM17 expression and activity in pathologic conditions, which corresponds to the increase of sACE2 concentration in plasma samples [30,51]. sACE2 concentration increases in patient cohorts with heart failure, mostly in males, and in patients with worse New York Heart Association functional class (NYHA) [18,51,52,53]. In patients with CVDs, the relation between the reduction of ACE2 protein on membranes and the susceptibility toward SARS-CoV-2 is still unclear. These patients could manifest a higher risk of adverse outcomes after SARS-CoV-2 infection because the virus uptake could worsen the pre-existing RAS disequilibrium [54]. Furthermore, patients with diabetes could have a higher susceptibility to SARS-CoV-2 infection because diabetes is frequently associated with an altered immune system [37]. Specifically, diabetic patients have delayed immune response and maladaptive inflammatory response as a consequence of the infection of β-cells by SARS-CoV-2 may aggravate clinical conditions [3,37] (Figure 3).

## 5. SARS-COV-2 Infection Starts from Lungs and Involves Heart

Airway epithelial cells are the first target of SARS-CoV-2 infection [29]. Severe cases of COVID-19 are frequently associated with multi-organ failure due to direct SARS-CoV-2 cytotoxicity and massive cytokines release [29,55,56,57]. It is suggested that classifying ACE2 organ distribution could be useful to understand SARS-CoV-2 pathogenesis [9]. ACE2 expression was detected in various organs such as the heart, lung, kidneys, oral cavity, brain, pancreas, gastrointestinal tract and brain [7]. Therefore, it is likely that organ vulnerability and severity of pathology depend on the level of ACE2 gene expression [7,9,58], aligning with the hypothesis that high ACE2 levels have been detected in both lungs and heart [9,16,59]. In addition, alveolar epithelial type II cells and cardiomyocytes (mostly aged ones) have high expression of genes that positively regulate viral reproduction and transmission thus, consistently with lung and heart vulnerability [22,60]. In contrast, the ileum is not the most vulnerable organ although it has the highest ACE2 expression [9]. This discrepancy requires further study. Additionally, alternative mechanisms of infection and organ susceptibility have been proposed such as ACE2 homodimers, co-receptor, and alternative receptors [9]. In ACE2 knockout mice, SARS-CoV-2 infection decreases but it is not completely prevented thus alternative host–virus interactions such as virus uptake in endosomes via cathepsin L need to be defined [9,26,61,62]. It is likely that multiple entry mechanisms might coexist, depending on target cell expression and pathological stages [9].

In severe COVID-19, it seems that early death occurs because of simultaneous respiratory failure and cardiac injury [36,55]. Autopsies on patients affected by COVID-19 with and without comorbidities clarified microscopic and macroscopic alterations in various organs after SARS-CoV-2 infection [63]. SARS-CoV-2 enhances classical RAS and may lead to multi-organ involvement and higher mortality. Interestingly, even patients without comorbidities manifest heart impairment [63] since patients admitted to intensive care units (ICU) have significantly increased levels of myocardial injury markers (i.e., creatine kinase-MB, high-sensitivity cardiac Troponin I) [7]. To date, the exact molecular mechanisms that correlate with myocardial injury following SARS-CoV-2 infection remain to be defined [64]. Current data indicate that cardiovascular injury in COVID-19 is probably of multifactorial origin. Specifically, ARDS and reduced pulmonary functionality are due to an excessive and uncontrolled immune response, which has destructive effects on vasculature and alveoli [7,65]. Consequently, ARDS leads to low oxygen saturation levels and low oxygen supply to organs causing hypoxia and oxidative stress, which alter their normal functionality. Severe hypoxia increases the probability of cardiovascular injury and mortality risk [6]. SARS-CoV-2 infection leads to systemic inflammation causing an increase in blood viscosity, endothelial dysfunction, activation of the coagulation cascade and atherosclerotic plaque rupture [57]. Autopsies of individuals with COVID-19 have shown the presence of macrophages infiltration in damaged tissues [66]. It is likely that the increased systemic levels of pro-inflammatory cytokines could explain the elevated macrophage infiltration [67]. However, it may be that myocardial injury is associated with other mechanisms than macrophages infiltration [67]. Myocyte necrosis could be due to lymphocytes infiltration [66,68] and direct SARS-CoV-2 cytotoxicity after cardiomyocyte infection occurring in cases of high viral load and longer exposure time [64]. Thus, SARS-CoV-2-associated myocardial damage could be due to increased cytokines, cell-mediated immune response and direct cardiomyocytes infection [64,67,69]. All these factors increase the probability of the formation of micro-thrombi and myocardial infarction.

Apart from immune response and direct cytotoxicity, the effects of SARS-CoV-2 on the heart may arise from RAS disequilibrium. Severe cardiovascular complications are frequently associated with Ang II accumulation because of ACE2 decrease [13]. In particular, Ang II induces the increase of cardiomyocyte contractility and converts cardiac fibroblasts to pro-fibrotic myofibroblasts, which enhance the production of RAS signaling component and TGF-β [12]. Additionally, Ang II exerts effects on endothelial cells, which secrete cytokines, and on inflammatory cells leading to their activation [12]. As a result, the previously described effects of Ang II combined with Ang II-mediated oxidative stress promote cardiac remodeling, leading to hypertrophy. Therefore, ACE2-virus internalization and ACE2 shedding in SARS-CoV-2 infection suggests that these alterations could exacerbate cardiovascular damage [7].

## 6. Is ACEI/ARB Therapy during SARS-COV-2 Infection Safe?

According to guidelines, ACEi/ARB therapy reduces mortality rates in patients with acute myocardial infarction, heart failure, hypertension and diabetes [70,71]. ACEi/ARB blocks the ACE/Ang II/AT1R axis thus limiting Ang II production and enhance ACE2 expression thus potentiating its positive effects [70,71]. Because of increased ACE2 expression in patients with chronic treatment with ACEi/ARB, it is suggested that these patients might have a higher risk of SARS-CoV-2 infection and severe COVID-19 [72]. On the other hand, therapy suspension may worsen chronic illness and reduce survival probability after SARS-CoV-2 infection. Considering SARS-CoV-2 perturbations on RAS equilibrium, there is a great interest in evaluating the necessity of ACEi/ARB therapy suspension or compliance in the context of SARS-CoV-2 infection. Numerous studies suggest that ACEi/ARB therapy does not influence negatively mortality or susceptibility to virus infection [73,74,75,76,77]. Furthermore, evidence of ACEi/ARB effectiveness is reported in publications based on meta-analysis as well [78,79]. The recent BRACE-CORONA trial suggests that treatment interruption does not positively affect the survival of COVID-19 patients, thus ACEi/ARB therapy should be continued [80]. On the contrary, a small number of studies do not recommend the use of ACEi/ARB. However, these studies suffer from limited cohort numbers that confound the interpretation and significance of the analysis [81,82]. Therefore, current guidelines of international and Italian scientific cardiovascular societies recommend not to interrupt ACEi/ARB treatment in patients with chronic comorbidities, even temporarily.

## 7. Do COVID-19 Vaccines Influence ACE2 Availability?

As of 7 April 2021, according to the WHO, there are 86 COVID-19 vaccines in clinical development and 186 under pre-clinical evaluation [83]. The Pfizer-BioNTech (BNT162b2), Moderna (mRNA-1273) and AstraZeneca ChAdOx1 nCoV-19 (AZD1222) vaccines are being rolled out currently [84,85]. All three vaccines promote humoral and cellular immune responses against S protein of SARS-CoV-2 [86,87]. At the present time, European Medicine Agency (EMA) is evaluating additional three COVID-19 vaccines: Russia’s Gamaleya National Centre of Epidemiology and Microbiology (Sputnik V), CureVac AG (CVnCoV) and Novavax CZ AS (NVX-CoV2373) [88].

In BNT162b2 and mRNA-1273, lipid nanoparticles (LNPs) carry nucleoside-modified RNA coding full-length S proteins with two proline mutations to keep it in the pre-fusion conformation [86,87,89]. Specifically, BNT162b2 administration consists of two doses of 30 µg within 21 days of each other, while two doses of 100 µg at the same time distance are needed for mRNA-1273 [90,91]. Clinical studies guarantee 95% and 94.1% efficacy for BNT162b2 and mRNA-1273 respectively [90,91]. On the other hand, AZD1222 uses a replication-incompetent chimpanzee adenovirus vector as a delivery vehicle for the wild-type version of S protein [92]. According to a pre-clinical trial on rhesus macaques, the main advantage of this strategy consists of inducing innate and adaptive immune response as in the case of viral infection [87]. The injection of 5 × 10^10^ viral particles leads to 70% of efficacy once the second dose is administrated after about 21 days within the first dose [92]. Recent events of thromboembolisms after AZD1222 administration indicated the necessity of further investigations on its safety. However, the EMA final report indicates that the vaccine is not associated with an increased risk of blood clotting [93].

Furthermore, among the COVID-19 vaccines under clinical evaluation, the Italian biotechnological company ReiThera developed the GRAd-COV2 vaccine based on replication-incompetent gorilla adenoviral vector encoding the full-length S protein [94]. This viral vector has advantages as low human exposure to gorilla adenovirus, high vector efficacy and possible strong immune response [94]. In light of the success of phase 1 trials approved by the Italian Medicine Agency (AIFA), the COVITAR trial is currently assessing phase two and three of clinical trials [94,95,96].

The aim of these strategies is to deliver nucleic acid, via LNPs or viral vector, which subsequently allows for S protein production in host cells. The presentation of peptides, from S protein, on class I and class II MHC, drives the humoral and cell-mediated immune response [97]. Studies confirm that vaccine administration leads to the production of neutralizing antibodies and robust CD8+ and CD4+ T-cell (Th1) response [90,91,92,98]. However, because BNT162b2 and mRNA-1273 are injected intramuscularly, it is likely that they do not induce strong mucosal immunity with the production of IgA to protect the upper respiratory tract [87]. Based on previous observations on SARS-CoV, S protein induces the reduction of ACE2 expression [99]. Notably, in a recent study, in vitro and in vivo analyses show that the S protein of SARS-CoV-2 could cause an ACE2 decrease as well [100]. However, it is still unknown if SARS-CoV-2 S protein expression in host cells could affect ACE2 availability on the cell surface after vaccination thus further investigations are needed.

## 8. Challenges

The unprecedented global efforts in developing safe and effective vaccines and making them available after rapid approval is an encouraging starting point to contain the explosive global spread of SARS-CoV-2. Apart from a regular supply of vaccines, prioritizing vaccine rollout and cold chain administration issues, several challenges remain. These include the effects of vaccines in older patients and those with comorbidities, as well as the long-term immunity benefits and efficacy on new South Africa, UK and Brazil SARS-CoV-2 variants [101,102]. To date, vaccination priority lists vary among nations depending on various socio-economic factors [101]. 

The discovery of new variants and slow vaccination campaigns might raise the risk of immune escape [103]. New emerging evidence has confirmed the worldwide presence of three main SARS-Cov-2 variants, 501Y.V1 (B.1.1.7), 501Y.V2 (B.1.351) and 501Y.V3 (P.1) respectively identified in The United Kingdom, South Africa and Brazil, which show immune escape thus, raising doubts about their influence on the effectiveness of current vaccines and hyper-immune serum. Hyper-immune serum obtained from the plasma of patients who had COVID-19 or animals, thereby inoculated with SARS-CoV-2 antigens, has been also authorized to successfully treat COVID-19 patients showing benefits in asymptomatic or symptomatic patients within three days [104,105]. Interestingly, the 501Y.V2 (B.1.351) viral variant confers partial to complete resistance to hyper-immune serum and reduces the efficacy of the AZD1222 vaccine [92] and sera from Pfizer-BioNTech and Moderna subjects displayed considerably decreased effects on 501Y.V2 [106]. As a consequence, pharmaceutical companies, proposing that changing the immunization sequence would cause a comparable neutralizing effect, trying to counteract the viral variants by replacing the original SARS-CoV-2 immunizing sequence with the 501Y.V2 sequences (i.e., Moderna) [107]. Therefore, these variants might be especially challenging in patients with cardiovascular diseases based on recent observations on animals [108].

Based on experience on vaccine production against other Coronaviruses some key points need further consideration [86]. Namely, hypersensitivity reactions driven by the high Th2 type response with eosinophil infiltration and abnormal antibody responses triggering the potential systemic breakdown are of particular importance [86]. However, it is still unknown if SARS-CoV-2 vaccines could have the same effects. To date, all studies of SARS-CoV-2 vaccines guarantee previously reported efficacy on specific age groups (16–55 years old BNT162b2; 18–65 years old mRNA-1273; 18–55 years old AZD1222) [90,91,92]. Furthermore, trials evaluating BNT162b2, mRNA-1273 and AZD1222 on elder individuals suggest that they are as safe as younger groups with the same antibody and cell response [91,109,110].

## 9. Conclusions

The intimate crosstalk between SARS-CoV-2 and ACE2 is even more complex than solely virus-receptor interaction. In fact, many pre-existing comorbidities originate from RAS misbalance causing higher susceptibility to virus or an increase of mortality risk after infection. Of note, it seems that death happens more frequently in case of cardiovascular injury. In fact, after SARS-CoV-2 infection, the virus mediates the worsening of clinical conditions because of its cytotoxicity, uncontrolled inflammatory response and RAS homeostasis loss. In this particular scenario, therapeutic approaches need close evaluation to avoid even more harmful effects. Contextually, ACEi/ARB is suggested as safe pharmacological therapy. In light of the recent vaccination campaign, further studies regarding the side effects of vaccines on pharmaceutical therapies and on ACE2 expression and physiological presence are also necessary. To date, ACEi/ARB treatment interruption in patients with chronic comorbidities appears unjustified. The rollout of COVID-19 vaccines provides opportunities to study their side effects and specific protective effects on ACE2 also in patients on treatment with ACEi/ARB.

## Abbreviations

ACE2	Angiotensin-converting enzyme 2
ACEi	Angiotensin-converting enzyme inhibitors
AIFA	Italian Medicine Agency
ARB	Angiotensin receptor blockers
ADAM17	ADAM metalloproteinase domain 17
Ang 1-7	Angiotensin 1-7
Ang 1-9	Angiotensin 1-9
Ang I	Angiotensin I
Ang II	Angiotensin II
ARDS	Acute respiratory distress syndrome
AT1R	Angiotensin type 1 receptor
AT2R	Angiotensin type 2 receptor
COVID-19	Coronavirus disease 2019
CVDs	Cardiovascular diseases
EGFR	Epidermal Growth Factor Receptor
EMA	European Medicine Agency
IL-6	Interleukin-6ICD Intracellular domain
MasR	Mas receptor
NYHA	New York Heart Association functional class
RAS	Renin-angiotensin system
RLRs	RIG-like receptors
sACE2	Soluble ACE2 fragment
SARS-CoV-2	Severe acute respiratory syndrome coronavirus 2
TLRs	Toll-like receptors
TMPRSS2	Type II transmembrane serine protease
TNF-α	Tumor necrosis factor alpha References
